# Identification of prognostic immune-related genes in rhabdoid tumor of kidney based on TARGET database analysis

**DOI:** 10.18632/aging.202475

**Published:** 2021-02-11

**Authors:** Huimou Chen, Suying Lu, Jinqiu Guan, Xiaoqin Zhu, Feifei Sun, Junting Huang, Jia Zhu, Juan Wang, Zijun Zhen, Yi Que, Xiaofei Sun, Yizhuo Zhang

**Affiliations:** 1 State Key Laboratory of Oncology in South China, Collaborative Innovation Center for Cancer Medicine, Guangzhou, Guangdong 510060, P.R. China; 2 Department of Pediatric Oncology, Sun Yat-sen University Cancer Center, Guangzhou, Guangdong 510060, P.R. China

**Keywords:** malignant rhabdoid tumor of kidney, TARGET database, immune-related genes, disease prognosis

## Abstract

Background: Malignant rhabdoid tumor of the kidney (RTK) is a rare and highly aggressive pediatric malignancy. Immune system dysfunction is significantly correlated with tumor initiation and progression.

Methods: We integrated and analyzed the expression profiles of immune-related genes (IRGs) in 65 RTK patients based on the Therapeutically Applicable Research to Generate Effective Treatments (TARGET) database. Prognostic related IRGs in RTK patients were analyzed using univariate and multivariate analysis, based on which a prognostic model with IRGs was constructed. Correlation analysis between the risk score of our model and tumor-infiltrating cell were also investigated.

Results: Twenty two IRGs were significantly associated with the clinical outcomes of RTK patients. Gene ontology (GO) analysis revealed that inflammatory pathways were most frequently implicated in RTK. A prognostic model was constructed using 7 IRGs (*MMP9, SERPINA3, FAM19A5, CCR9, PLAUR, IL1R2, PRKCG*), which were independent prognostic indices that could differentiate patients based on their survival outcomes. Furthermore, the risk scores from our prognostic model was positively associated with cancer-associated fibroblasts (CAFs).

Conclusions: We screened seven IRGs of clinical significance to distinguish patients with different survival outcomes. This may enhance our understanding of the immune microenvironment of RTK, and could use to design individualized treatments for RTK patients.

Background: Malignant rhabdoid tumor of the kidney (RTK) is a rare and highly aggressive pediatric malignancy. Immune system dysfunction is significantly correlated with tumor initiation and progression.

Methods: We integrated and analyzed the expression profiles of immune-related genes (IRGs) in 65 RTK patients based on the Therapeutically Applicable Research to Generate Effective Treatments (TARGET) database. Prognostic related IRGs in RTK patients were analyzed using univariate and multivariate analysis, based on which a prognostic model with IRGs was constructed. Correlation analysis between the risk score of our model and tumor-infiltrating cell were also investigated.

Results: Twenty two IRGs were significantly associated with the clinical outcomes of RTK patients. Gene ontology (GO) analysis revealed that inflammatory pathways were most frequently implicated in RTK. A prognostic model was constructed using 7 IRGs (*MMP9, SERPINA3, FAM19A5, CCR9, PLAUR, IL1R2, PRKCG*), which were independent prognostic indices that could differentiate patients based on their survival outcomes. Furthermore, the risk scores from our prognostic model was positively associated with cancer-associated fibroblasts (CAFs).

Conclusions: We screened seven IRGs of clinical significance to distinguish patients with different survival outcomes. This may enhance our understanding of the immune microenvironment of RTK and could use to design individualized treatments for RTK patients.

## INTRODUCTION

Malignant rhabdoid tumor of the kidney (RTK) is a rare and highly lethal malignancy that primarily affected infants and young children [1]. Previous studies have reported that 10–15% of patients with RTKs had primary central nervous system (CNS) disease, which is currently designated as atypical teratoid-rhabdoid tumors [2]. The morbidity associated with RTK is extremely rare, accounting for about 2% of renal tumors in children. The prognosis of RTK patients remains to be extremely poor, with an overall 5-year survival rate of no more than 20% to 25% [3–5]. The main obstacle to improve the survival rate of patients with RTK might be due to poor understanding of the biological characteristic and regulatory mechanisms that underlie this fatal disease.

Recently, increasing evidence has shown that immune system dysfunction might play a crucial role in tumorigenesis and progression of cancer [6]. Several studies have proved the association between programmed death-1 (PD-1) or cytotoxic T-lymphocyte associated antigen 4 (CTLA-4) polymorphisms and the immune escape of cancer. Immune checkpoint inhibitors (ICIs), such as pembrolizumab and nivolumab, have shown apparent efficacy in reducing tumor growth. This is mainly done by interfering with PD-1 or programmed death ligand-1 (PD-L1) interaction, thereby limiting the immune escape of cancer cells [7]. Also, certain ICIs have been shown to greatly improve the prognosis of patients [8–11]**.** However, only a small subset of patients responds to these therapies. Therefore, it is urgent to screen patients who are sensitive to immunotherapy [9].

Accumulating studies have suggested that immune-related genes (IRGs) showed association with the efficacy of immunotherapy and the prognosis of patients [10]. According to a previous study, high OX-40 expression in tumor immune infiltrates indicated a favorable prognosis in patients with non-small cell lung cancer [11]. Zhang et al. have reported that PKD1 is significantly overexpressed in the tissues and remarkably associated with dismal prognosis in osteosarcoma [12]. Ryu et al. have found that the expression of p16 showed association with tumor immune microenvironment and had favorable prognosis of head and neck squamous cell carcinoma [13]. Besides, Bai et al. have revealed that the expression of BRAF V600E showed positive association with PD-L1/PD-1 in papillary thyroid carcinoma (PTC) samples, suggesting that immunotherapies that target PD-L1/PD-1 might be effective in PTC patients with BRAF V600E mutation [14]. However, there are only few studies that focused on the roles of IRGs in RTK [15]. Therefore, in this study, the Therapeutically Applicable Research To Generate Effective Treatments (TARGET) database were searched to examine the relationships of multiple immune genes with the prognosis of RTK, and aimed to construct a new prognostic model of RTK with IRGs, verifying the validity of this model in RTK patients based on TARGET-RTK cohort.

## RESULTS

### DEGs from RTK and normal controls

**Figure 1:**
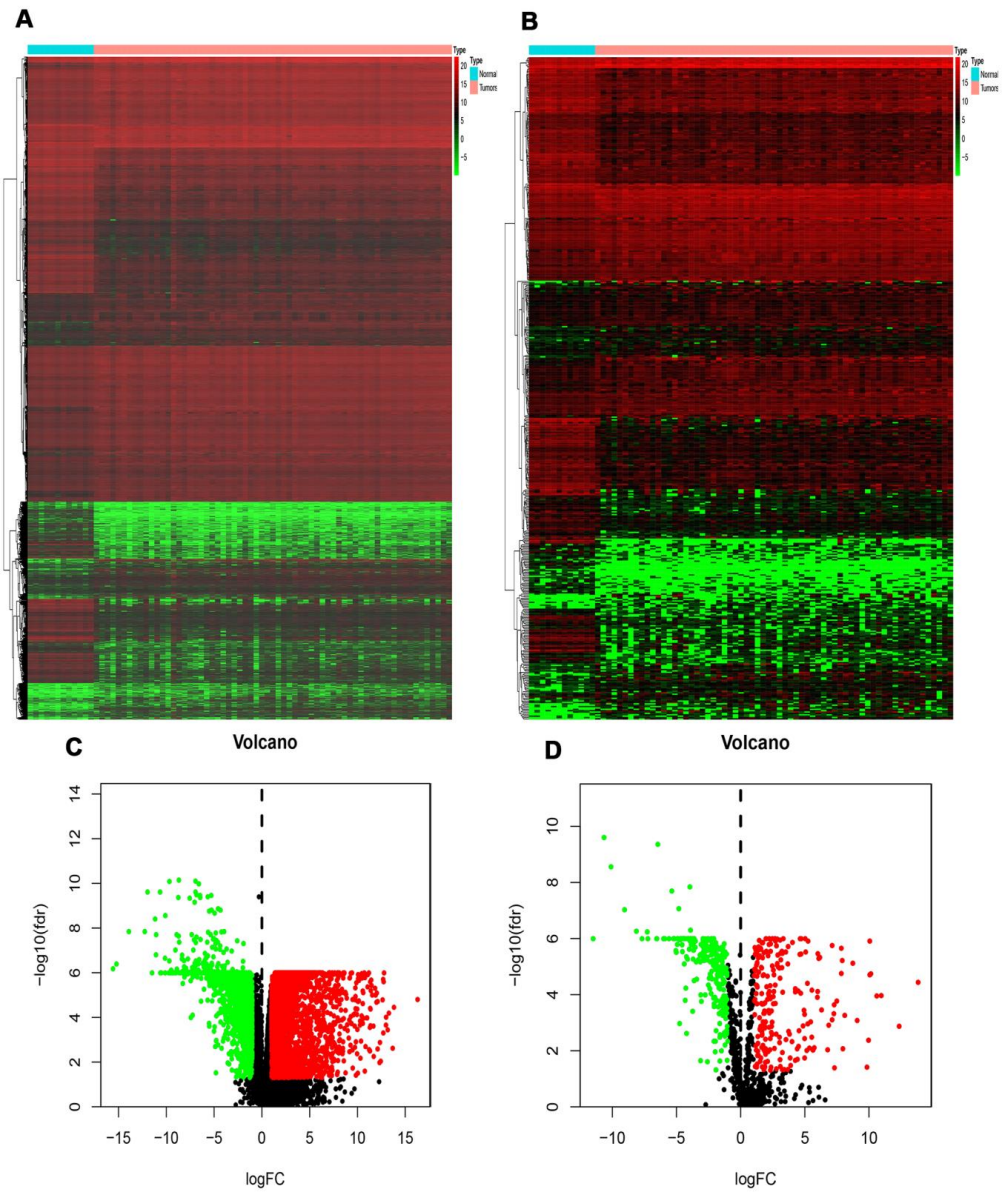
**Differentially expressed genes and IRGs in RTK.** (**A**) Heat-map of significant DEGs in RTK. The color from green to red represents the progression from low expression to high expression. (**B**) Heatmap of significant differentially expressed immune-related genes in RTK. Red represents higher expression while green represents lower expression. (**C**) Volcano plot of differentially expressed genes. The red dots in the plot represents up-regulated genes and green dots represents down-regulated genes with statistical significance. Black dots represent no DEGs. (**D**) Volcano plot of differentially expressed immune-related genes in RTK. Colored dots represent differentially expressed immune-related genes and black dots represent no differentially expressed immune-related genes. Abbreviations: GO, Gene Ontology. IRGs, immune-related genes. DEGs, differentially expressed genes.

In this study, 7002 DEGs (3927 up-regulated genes and 3075 down-regulated genes) were found in the RTK samples when compared with normal control samples. They had a | log FC ≥ 1 and a false discovery rate (FDR) < 0.05. The heatmap and volcano diagrams of all DEGs are shown in Figure 1A, 1C.

### Identification of DEIRGs

**Figure 2:**
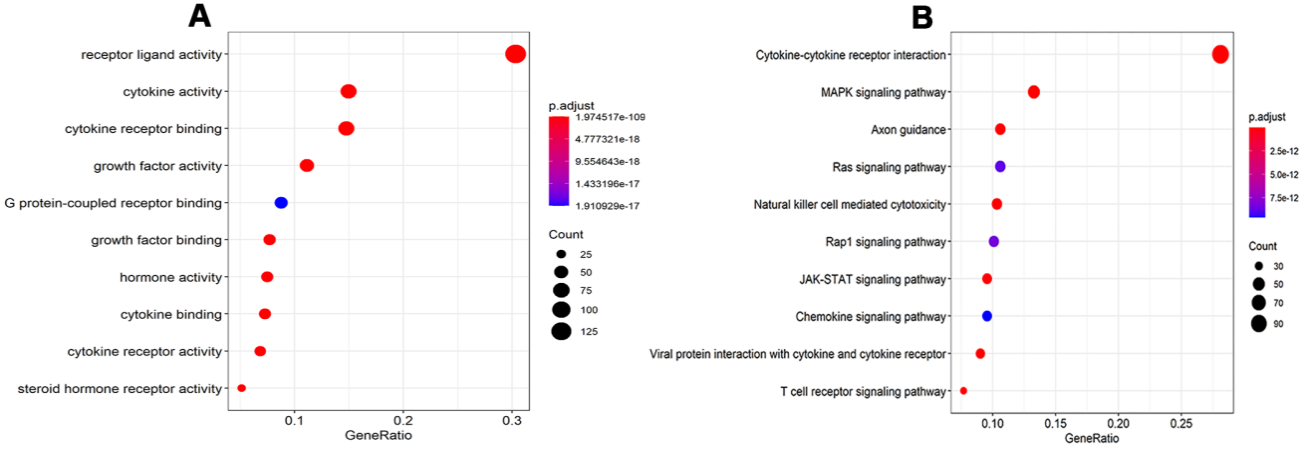
**GO analysis and KEGG pathways of IRGs.** (**A**) GO analysis of differentially expressed IRG. (**B**) KEGG pathways of IRGs. Abbreviations: GO, Gene Ontology. KEGG, Kyoto Encyclopedia of Genes and Genomes. IRGs, immune-related genes.

A total of 478 DEIRGs, including 254 up-regulated IRGs and 224 down-regulated IRGs, were extracted from the DEGs. The heatmap and volcano diagrams of all DEIRGs were shown in Figure 1B, 1D. As expected, the GO analysis revealed that inflammatory pathways were the most frequently implicated ones, and the receptor-ligand activity, cytokine activity, and cytokine receptor binding, growth factor activity, and cytokine receptor activity were the most frequent biological processes in RTK (Figure 2A). Based on the KEGG pathways analysis, our results demonstrated that DEIRGs were particularly enriched in cytokine receptor interactions of signaling pathways (*p*< 0.001), (Figure 2B).

### Identification of prognostic DEIRGs

As RTK is a lethal disease, monitoring the disease outcomes is important for clinical management. So, the molecular biomarkers that could serve as viable prognostic indicators were identified. After univariate Cox regression analysis, 22 DEIRGs were shown to be significantly associated with the OS of RTK patients in the TARGERT cohort (*p*< 0.05), (Table 1).

**Table 1:** Univariate regression analyses of prognostic IRGs for overall survival.

**Gene**	**HR(95%CI)**	**P**
PLAUR	1.637(1.183-1.922)	0.001113
NR0B2	1.30(1.076-1.538)	0.001467
GHR	1.379(1.056-1.679)	0.003304
SERPINA3	1.192(1.0045-1.316)	0.004133
GFAP	1.217 (1.145-1.362)	0.005889
HRG	1.170(1.098-1.236)	0.006228
PRKCG	1.204(1.123-1.301)	0.011821
IL1R	1.275(1.034-1.413)	0.012937
OXT	1.278(1.091-1.386)	0.016251
TRBV6-5	0.838(0.273-0.903)	0.017914
ANGPTL6	1.249(1.074-1.373)	0.018051
CCR9	0.792(0.376-0.895)	0.020727
S100A14	1.161(1.063-1.317)	0.022779
PLTP	1.655(1.176-1.803)	0.029601
FCN2	1.201(1.026-1.415)	0.03082
F2RL1	1.148(1.005-1.227)	0.03488
MMP9	0.865(0.212-0.987)	0.036752
AZGP1	1.149(1.101-1.237)	0.039613
ARTN	1.284(1.078-1.382)	0.042109
DEFB1	1.122(1.002-1.207)	0.048537
AVP	1.436(1.014-1.566)	0.049212
FAM19A5	0.814(0.348-0.893)	0.04268

Abbreviations: IRGs, immune-related genes. HR, hazard ratio. CI, confidence interval.

### TF (transcription factor) regulatory network

**Figure 3:**
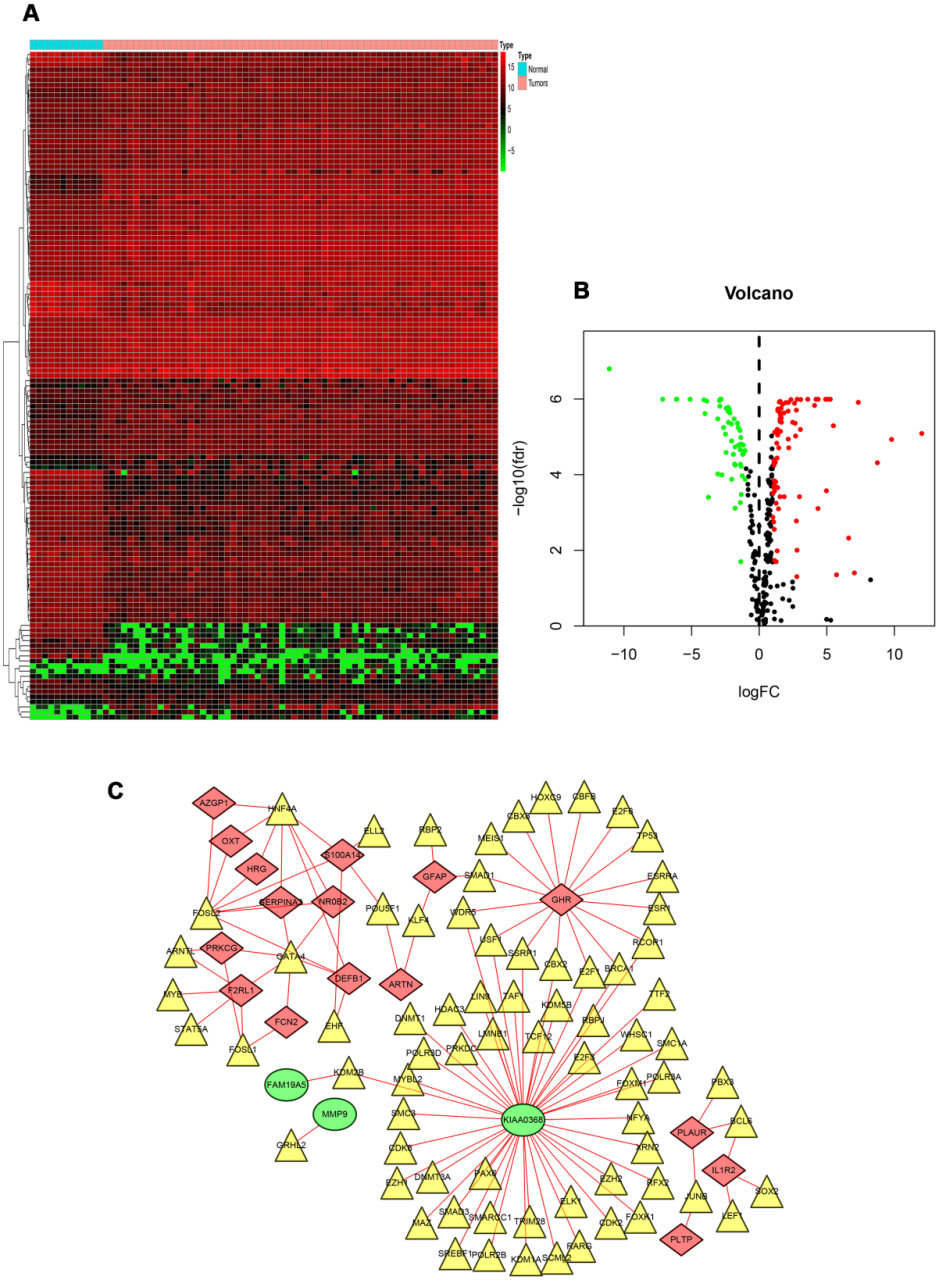
**TF-based regulatory network.** (**A**) Heat map of differentially expressed TFs. The green to red spectrum indicates low to high TF expression. (**B**) Volcano plot of TFs. The green dots represent down-regulated TFs, the red dots represent up-regulated TFs and the black dots represent TFs that were not significantly and differentially expressed. (**C**) Regulatory network of TFs and PIRGs; the green nodes represent PIRGs with hazard ratios of <1 (*p* < 0.05), the red nodes represent PIRGs with hazard ratios of >1 (*p* < 0.05), the yellow nodes represent TFs that were correlated with PIRGs in terms of their mRNA levels (correlation coefficient > 0.4 and *p* < 0.01), and the red lines indicate positive regulatory relationships. Abbreviations: TF, transcription factor. PIRGs, prognostic immune-related genes.

To explore the potential regulatory mechanisms that correspond to the clinical significance of DEIRGs, the correlation between TFs and PIGRs (prognostic related immune genes) was analyzed. Firstly, the mRNA levels of TFs in RTK samples and normal controls were analyzed. A total of 131 differentially expressed TFs were identified between the two tissue types (FDR < 0.05, |log2 FC| > 1). Next, the correlations between the mRNA levels of the 131 TFs and the 22 PDEIRGs, with a correlation coefficient > 0.4 and a *p*-value < 0.01 as the cut-off values were identified (Figure 3A, 3B). A total of 73 TFs were shown to be significantly associated with the abnormal expression of PIRGs (*P*< 0.01). To better visualize the regulatory relationships, a TF-based regulatory network was constructed using the Cytoscape software Figure 3C. Among these, the three transcription factors including HNF4A, FOSL2, and GATA4 interacted with multiple immune-related genes.

### Construction of immune-related genes prognostic model for RTK

Multivariate Cox regression analysis identified 7 genes that showed significant association with the clinical outcomes (Table 2), which were used to construct a prognostic model to separate the RTK patients into two groups with discrete clinical outcomes. The formula was as follows:

**Table 2:** Multivariate regression analyses of prognostic IRGs for overall survival.

**Gene**	**Coef**	**HR(95%CI)**	***P***
MMP9	-0.32924	0.719(0.622-0.816)	0.000691
SERPINA3	-0.13156	0.876(0.795-0.958)	0.106496
FAM19A5	-0.46497	0.628(0.493-0.763)	0.000571
CCR9	-0.40906	0.664(0.561-0.766)	6.56E-05
PLAUR	0.603828	1.829(1.638-2.019)	0.001552
IL1R2	0.476825	1.610(1.467-1.754)	0.000907
PRKCG	0.365902	1.441(1.337-1.546)	0.000457

Abbreviations: IRGs, immune-related genes. HR, hazard ratio. CI, confidence interval. Coef, coefficient.

[Expression level of MMP9 *(-0.32924)] + [Expression level of SERPINA3 * (-0.13156)] + [Expression level of FAM19A5 * (-0.46497)] + [Expression level of CCR9 * (-0.40906)] +[Expression level of PLAUR * 0.603828] + [Expression level of IL1R2 * 0.476825]+ [Expression level of PRKCG * 0.365902].

**Figure 4:**
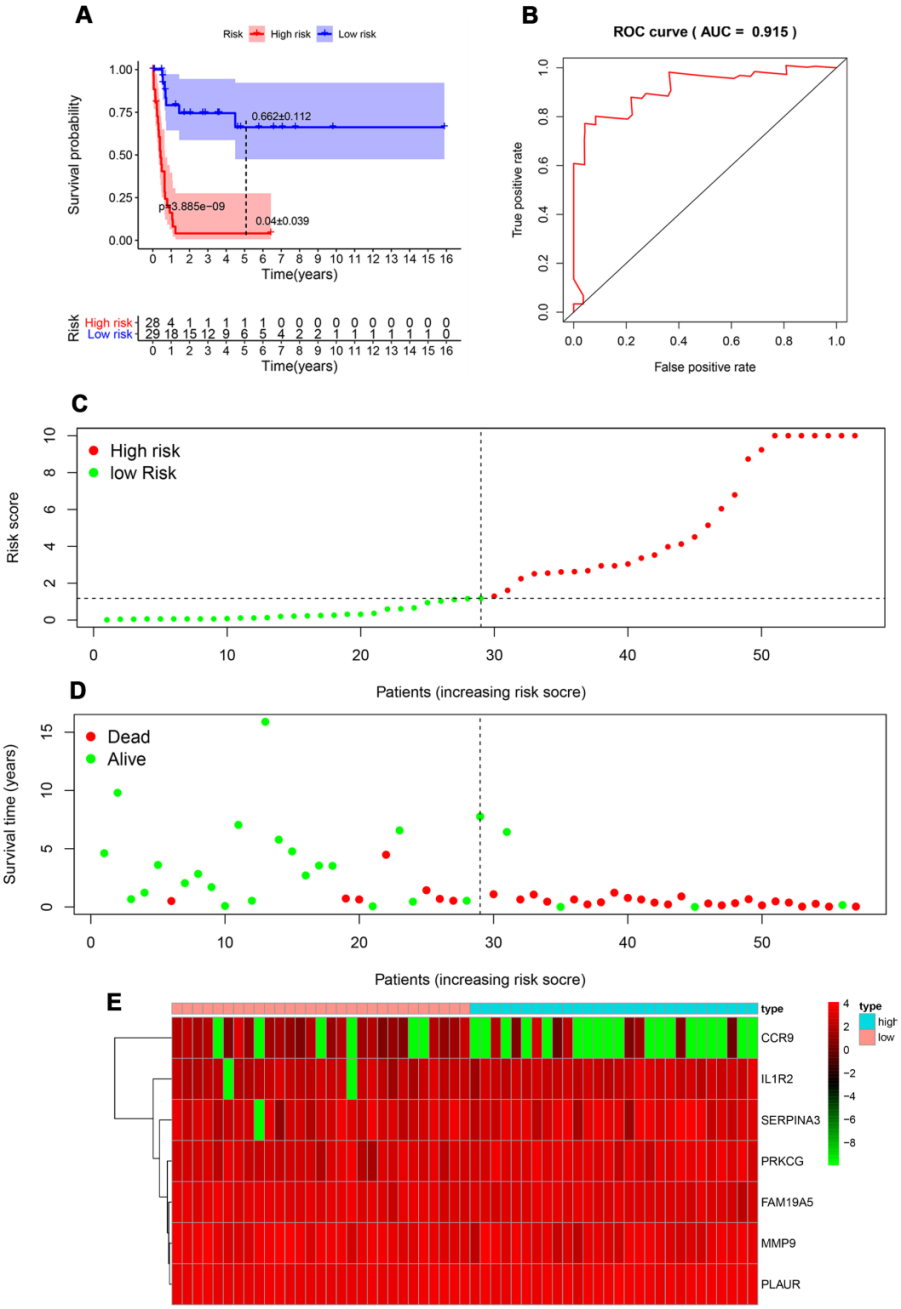
**Prognostic analysis of the TARGET-RTK cohort.** (**A**) Kaplan-Meier curve analysis of the high-risk and low-risk groups. (**B**) Survival-dependent receiver operating characteristic (ROC) curve validation of the prognostic value of the prognostic index. (**C**) Dot plot of the risk score. Vertical and horizontal axes represent risk score and RTK samples, ranked by increasing risk score. Red and green colors represent high-and low-risk cases, respectively. (**D**) Dot plot of survival. Vertical and horizontal axes represent the survival times and RTK samples, ranked by increasing risk score. Red and green colors represent dead and living RTK cases, respectively. (**E**) Heat map of the expression levels of the seven genes. Vertical and horizontal axes represent genes and RTK samples, ranked by increasing risk score. Genes with higher, lower, and same expression levels are shown in red, green, and black, respectively. Color bars at the top of the heat map represent sample types, with pink and blue indicating low- and high-risk score samples, respectively. Abbreviation: RTK, rhabdoid tumor of kidney.

The median value of the risk scores was used as the cutoff value to divide patients in the TARGET-RTK cohort into high-risk group (n =28) and low-risk group (n = 30). As shown in Figure 4A, the number of deaths was significantly greater while the OS was shorter in high-risk cases when compared to the low-risk group (*p*< 0.001). The 1-year OS rate in the high-risk group of TARGET-RTK cohort was 16.5%, while the corresponding rate for that of the low-risk group was 79.2%. The area under the ROC (AUC) value for the prognostic model was 0.915, which suggested an excellent potential for the prognostic model based on PIRGs in monitoring the survival (Figure 4B). The risk scores of the patients in the TARGET-RTK cohort were ranked and then their distribution was analyzed (Figure 4C). The survival status of each patient in the TARGET-RTK cohort was marked on the dot plot in Figure 4D, showing the heatmap of the expression patterns of the risk genes in the two prognostic groups (Figure 4E).

### Independent prognostic value of the risk model in the TARGET-RTK cohort

**Figure 5:**
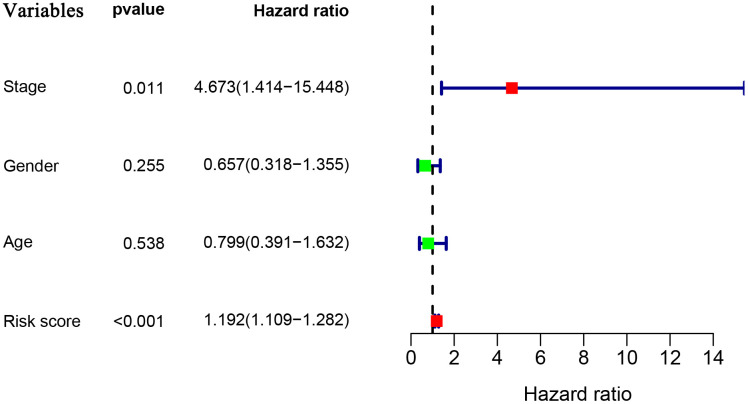
**Univariate Cox regression analyses in the entire TARGET cohort.**

**Figure 6:**
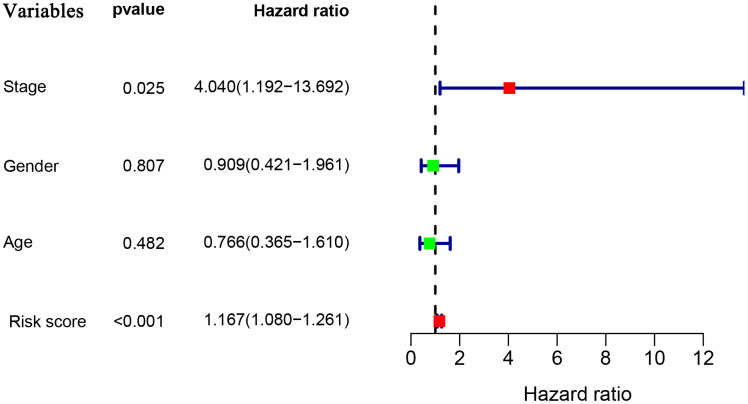
**Multivariate Cox regression analyses in the entire TARGET cohort.**

To explore whether the prognostic model acts as an independent prognostic index in RTK patients, univariate and multivariate Cox regression analyses were performed to assess the effectiveness of this model. As expected, univariate analyses indicated that the variables of the prognostic model and clinical stage showed significant association with the prognosis of RTK patients (Figure 5). Multivariate analysis revealed that the prognostic model was independently associated with the OS in the TARGET-RTK cohort (*p*< 0.001), (Figure 6). These results indicated that the prognostic model could be used independently for predicting the prognosis of RTK patients. Consistent with the clinical observation, the clinical-stage also acts as an independent prognostic indicator for RTK in multivariate analysis (*p*< 0.05).

### Clinical utility of the prognostic model

**Figure 7:**
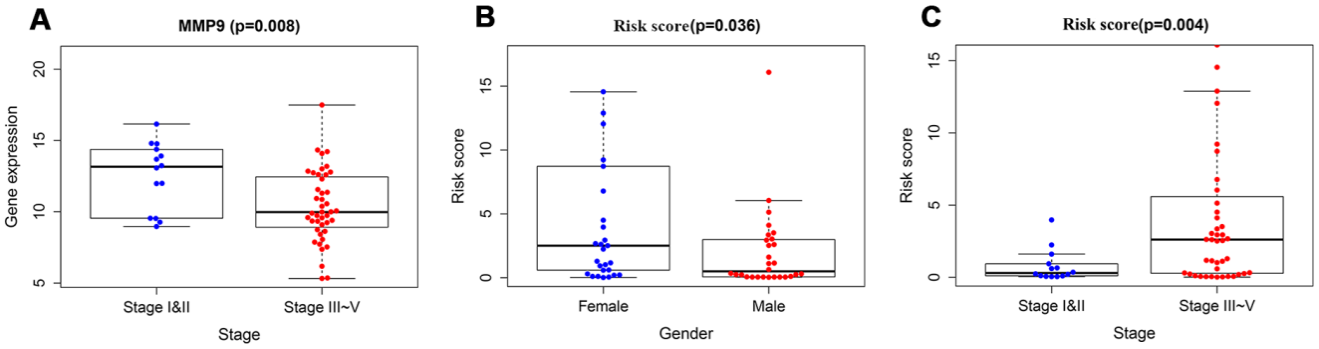
**The relationships between immune-based prognostic model and clinical and demographic characteristics.** (**A**) The relationships between the expression of MMP9 and stage. (**B**) The relationships between the risk scores and gender. (**C**) The relationships between the risk scores and stage.

To validate the clinical utility of our model in predicting the progression of RTK, the relationships were analyzed between our model (the risk score and risk genes) and clinical and demographic characteristics (age, gender, stage). The expression of MMP9 was lower in the advanced stage cases, while the risk scores were significantly higher in the advanced stage cases and female patients (Figure 7), and no difference was observed in other variables.

### Tumor-infiltrating cell

**Figure 8:**
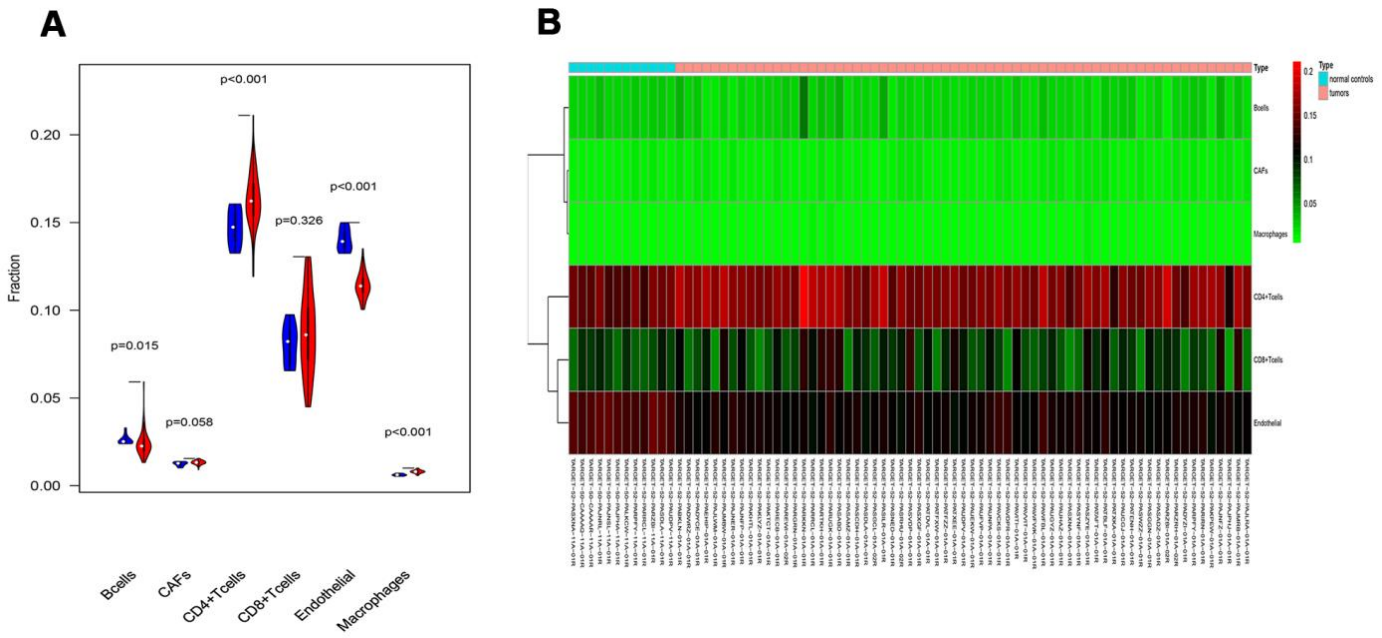
**Analysis of different tumor-infiltrating cells in the TARGET-RTK cohort.** (**A**) Violin plot comparing the proportions of TICs between normal and RTK samples. Horizontal and vertical axes represent TICs and relative percentages. Blue and red colors represent normal and tumor samples, respectively. Data were assessed by Wilcoxon rank-sum test. (**B**) Heat map of different TICs in the TARGET-RTK cohort. Abbreviation: TIC, tumor-infiltrating cell.

**Figure 9:**
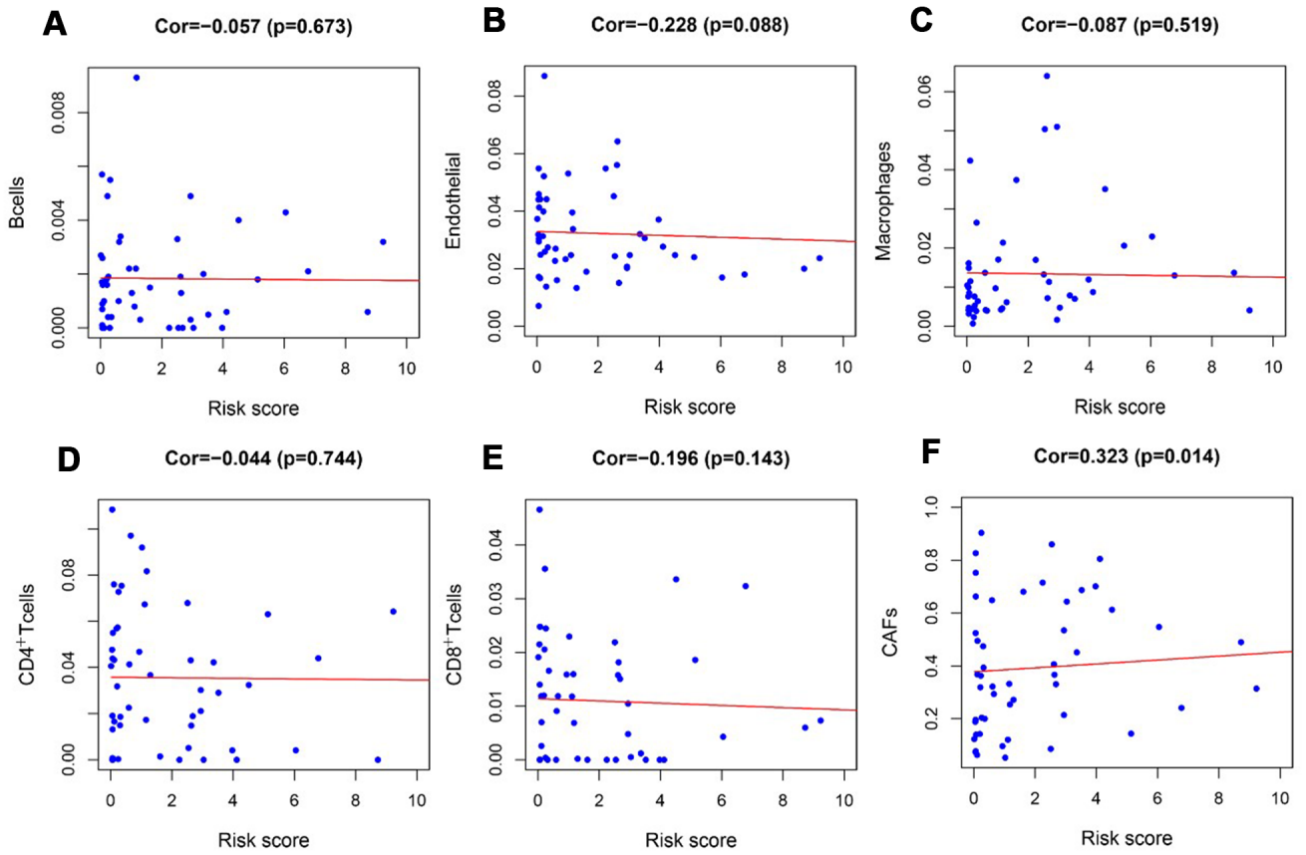
**Analysis of the correlation between the risk score and tumor-infiltrating cells in the TARGET-RTK cohort.** (**A**) B cells; (**B**) endothelial cells; (**C**) Macrophages. (**D**) CD4+ T cells. (**E**) CD8+ T cells. (**F**) CAFs. Abbreviation: CAFs, carcinoma-associated fibroblasts.

To verify whether our prognostic index model could reflect the status of the tumor immune microenvironment in RTK patients, the relationships between the risk score and tumor-infiltrating cells in the TARGET-RTK cohort were analyzed. There were fewer B cells, CD4^+^ T cells, CD8^+^ T cells, and endothelial cells in the tumor samples, while cancer-associated fibroblasts (CAFs) and macrophages were more in the tumor samples than in the normal controls (Figure 8). Furthermore, correlation analysis showed that the risk score was positively associated with CAFs, and no difference was observed between the risk score and other infiltrating cells (Figure 9).

## DISCUSSION

In the present study, integrated analysis of the TARGET-RTK cohort was conducted and the results revealed that 478 IRGs showed aberrant expression in RTK patients when compared to normal controls. Next, univariate and multivariate COX regression analyses were performed to explore the PIRGs, and the results showed that a total of seven PIRGs were used to construct an independent prognostic model. As expected, compared with the low-risk group, the number of deaths in the high-risk group was significantly greater, and the OS time was shorter. Furthermore, the expression profiles of tumor-infiltrating cells in RTK were analyzed. The results showed that there were more CAFs and macrophages in the tumor tissues than in the normal controls, while B cells, CD4^+^T cells, CD8^+^T, and endothelial cells were fewer than normal controls. Also, the relationship between the risk score and tumor-infiltrating cells was explored, and our results showed that the risk score was positively correlated with infiltrating CAFs.

Cancer research has uncovered it as a disease that involves a succession of alterations to the genome [16]. Our study explored the alterations to reveal the relationship between immunogenomic profiles and immune microenvironment and to uncover the potential clinical implications. GO analysis and KEGG indicated that these altered IRGs were mainly involved in cytokine-cytokine receptor interactions, *MAPK*, and *PI3K-Akt* signaling pathways. Previous studies have revealed that cytokine and their receptors actively participate in various types of cancers, including RTK [17, 18]. Some of these cytokines have been demonstrated to exert anti-tumor or tumor-promoting effects on rhabdoid tumor cell lines [18]. As such, these altered IRGs might serve as diagnostic markers or therapeutic targets for RTK. However, comprehensive and an in-depth exploration with regard to the role of these IRGs in RTK is needed. The results of KEGG analysis indicated that *PI3K-Akt* and *MAPK* signaling pathways were active in RTK, which were consistent with that of the previous studies [19, 20]. We hypothesized that inhibitors of *PI3K-Akt* and *MAPK* signaling pathways might be effective in RTK. Experiments and clinical trials are needed to further verify this conjecture.

Previous studies have revealed that TFs play an important role in the development and differentiation of immune cells and immune response by regulating IRGs [21, 22]. In our study, 73 TFs in the transcription factor network interacted with PIRG, indicating that these PIRGs might be regulated by these 73 TFs in RTK. Among these, three TFs including HNF4A, FOSL2, and GATA4 interacted with multiple IRGs. Increasing studies have demonstrated that HNF4A, FOSL2, and GATA4 showed aberrant expression in various malignant tumors, playing a role in promoting or inhibiting malignancies [8, 23–25], and our study results were consistent with these studies. We hypothesized that the abnormal expression of these three TFs might be crucial for the development and progression of RTK. However, there might exist other underlying regulatory mechanisms that are independent of these TFs. The molecular regulatory mechanism of these IRGs requires further research.

Previous studies analyzing the prognostic factors are limited because of the low incidence of RTK. Tomlinson G et al. have found that young age at diagnosis showed association with dismal survival of RTK patients [5]. However, little is known about the clinical importance of IRGs in RTK. In the present study, 7 IRGs that were closely associated with the clinical outcomes of children with RTK were found, and a prognostic model was constructed by using these to accurately discriminate between patients with different survival outcomes. Moreover, the risk scores from our prognostic model were significantly higher in advanced stage cases and female patients. It is well known that the prognosis of RTK patients with advanced-stage remained very poor, and our results were consistent with the clinical observation [26]. However, multivariate Cox regression analysis indicated that gender was not an independent prognostic factor. This inconsistency might be attributed to the small sample size. Based on our research, we hypothesized that this model could be applied to identify high-risk RTK patients, enabling early, intensive interventions and regular monitoring of disease recurrence to improve the prognosis of patients. Moreover, as revealed in the previous studies that tumor-infiltrating immune cells are important determinants of the prognosis and response to therapy [27, 28]. Our results indicated that ICIs might be effective for low-risk RTK patients based on our model. The comprehensive and integrated analyses of IRGs in RTK deepens our understanding with regard to their clinical significance and illuminates the underlying regulatory mechanism of these IRGs. There were several advantages in our study over other reports. Firstly, the ImmPort database used in this study was a specialized immunology database, allowing us to analyze as many IRGs as possible. Secondly, although previous studies have demonstrated that IRGs could be applied to predict the prognosis of patients with cancer, this is the first study to our knowledge that used such a great number of samples to comprehensively explore the clinical importance of IRGs in RTK patients. Thirdly, the novel immune-related prognostic model exhibited a prominent performance for predicting the OS based on the TARGET database. To our knowledge, this is the first prognostic model developed for RTK patients.

Studies have revealed that regulation of tumor immune microenvironment plays a crucial role in tumor progression and metastasis [29, 30]. To characterize the tumor immune microenvironment status, tumor-infiltrating immune cells were investigated. There were more CAFs and macrophages, but fewer B cells, CD4^+^T cells, CD8^+^T cells, and endothelial cells in the tumor tissues than normal controls. In addition to the difference in the number of tumor-infiltrating cells, the relationships between tumor-infiltrating cells and risk scores were further evaluated. Also, CAFs in the tumor stroma were found to be positively correlated with risk score, but no difference was observed between the risk score and other infiltrating cells. This might be due to relatively high content of CAFs and fewer tumor-infiltrating cells in RTK tissues. Many studies have suggested a prominent functional role of CAFs in cancer progression and metastasis over the past few years. CAFs in tumor microenvironment induce angiogenesis by secreting CAF derived stromal cell-derived factor 1 and recruiting bone marrow-derived endothelial cells to promote tumor growth [31, 32]. CAFs prompted epithelial to mesenchymal transition (EMT) and invasiveness of adjacent cancer cells by producing ECM-degrading proteases such as the MMPs [33]. CAFs promoted immunosuppressive TME, which is conducive to the immune escape of tumor cells [34]. Our result revealed that CAFs might play a tumor-promoting role in RTK, and this was consistent with the function of CAFs in other tumors [35, 36]. However, the specific role and regulatory mechanism of CAFs in RTK should be further confirmed by experiments.

Admittedly, our study also had some limitations. Firstly, the number of RTK tissue samples in the TARGET cohort was relatively small, which might in turn cause selection bias. Secondly, transcriptomic analysis could not reflect the global alterations of immune status. Thirdly, the prognostic model we constructed was not validated in the prospective clinical trials. Additionally, the reliability of IRGs identified requires further validation in both *in vivo* and *in vitro* experiments.

In conclusion, a prognostic model using 7 IRGs that showed significant correlation with clinical outcomes was constructed. The risk score acts as an independent prognostic index that could distinguish patients with different survival outcomes, and this could be applied to predict patients who were sensitive to immunotherapy.

## MATERIALS AND METHODS

### Database

The required original data were obtained from TARGET (https://ocg.cancer.gov/programs/target) database. A total of 77 samples were screened, which included 65 tissues from RTK patient and 12 samples of normal kidney tissues. All data were processed using the R software (https://www.r-project.org/) and normalized using the limma package.

### Analysis of differentially expressed genes (DEGs) by R software

DEGs were identified using the limma package [37]. The absolute value of | log2 fold change (FC) | was set to >1, and the cutoff value was adjusted to a *P*-value of < 0.05, which was considered to be statistically significant. The FDR and *P* values were screened to obtain all DEGs. The ggplot2 and the heatmap package were used to plot volcano diagrams and heatmap of DEGs, respectively.

### Identification of differential expression of immune-related genes (DEIRGs)

A total of 2498 IRGs were retrieved from the ImmPort database (https://www.immport.org/home) [38]. The DEIRGs were obtained from the DEGs using the R software. The absolute value of | log2 fold change (FC) | was set to >1, and the *P*-value was adjusted to <0.05 as cut-offs for filtering the DEIRGs. The ggplot2 and the heatmap package were used to plot the heatmap and volcano diagrams of the DEIRGs, respectively.

### Univariate and multivariate analysis of the DEIRGs

To explore the prognostic significance of each DEIRG, univariate Cox analysis was conducted to screen the IRGs with a significant prognostic value (*P*< 0.05) as the candidates of individual risk score. A multivariate Cox analysis was performed to identify robust IRGs (*P*< 0.05), showing association with the overall survival (OS) to build the IRGs prognostic model. The forest map was drawn with ggplot2 package.

### Molecular characteristics of prognostic immune-related genes (PIRGs)

DEIRGs that showed significantly relation to the OS of RTK patients were regarded as PIRGs. It is well known that the transcription factors (TFs) act as important biological regulators of gene function in response to various internal and external stimuli. To better understand the regulatory mechanism of these immune genes, it is necessary to identify some TFs in patients with RTK that have the potential ability to regulate these PIRGs. Cistrome Cancer is a comprehensive resource for predicting the targets and enhancer profiles of TFs in cancers. This database contains a total of 318 TFs, and these are considered as a precious resource for experimental and computational cancer biology research. After that, clinically relevant TFs were extracted to construct the regulatory network of clinically relevant IRGs and potential TFs.

### Generation of individual risk score

To assess the risk in each patient, a new prognostic model based on the expression data by multiplying with the Cox regression coefficient was constructed. The prognostic model was used to measure the prognostic risk of each patient with RTK, and the median risk score of the cohort was used as the cut-off value to divide all RTK patients into two groups: the high and low-risk groups. High-risk score indicates a poor prognosis in RTK patients.

### Analysis of tumor-infiltrating cell

EPIC is an online database that is designed to estimate the proportion of immune and cancer cells from bulk tumor gene expression data (https://gfellerlab. shinyapps.io/EPIC_1-1/) [39]. After analyzing the immune infiltrating cells, including B cells, CAFs, CD4^+^ T cells, CD8^+^T cells, endothelial, macrophages, and natural killer (NK) cells, the correlation between the risk score and tumor-infiltrating cells was analyzed by the R software.

### Statistical analysis

The R software cluster Profiler package was used to perform GO analysis for identifying the biological themes among the gene clusters. The Kyoto Encyclopedia of Genes and Genomes (KEGG) was used to screen the pathway enrichment of IRGs using the cluster Profiler package. The survival ROC R software package was used to calculate the AUC of the survival ROC curve to measure the performance of the prognostic model. The differences among the clinical parameters were tested using independent t-tests. R software was used to perform all statistical analyses, and *p*< 0.05 was considered to be statistically significant.
